# Molecular Insights into FaEG1, a Strawberry Endoglucanase Enzyme Expressed during Strawberry Fruit Ripening

**DOI:** 10.3390/plants8060140

**Published:** 2019-05-28

**Authors:** Karla Jara, Ricardo I. Castro, Patricio Ramos, Carolina Parra-Palma, Felipe Valenzuela-Riffo, Luis Morales-Quintana

**Affiliations:** 1 Instituto de Ciencias Biológicas, Universidad de Talca, Talca 3465548, Chile; karla.jara@utalca.cl (K.J.); pramos@utalca.cl (P.R.); cparra@utalca.cl (C.P.-P.); 2 Multidisciplinary Agroindustry Research Laboratory, Instituto de Químicas Aplicadas, Universidad Autónoma de Chile, Talca 3460000, Chile; ricardo.castro@uautonoma.cl; 3 Núcleo Científico Multidiciplinario-DI, Universidad de Talca, Talca 3465548, Chile; 4 Multidisciplinary Agroindustry Research Laboratory, Instituto de Ciencias Biomédicas, Facultado de Ingeniería, Universidad Autónoma de Chile, Talca 3460000, Chile

**Keywords:** endoglucanase, cell wall disassembly, molecular modeling, molecular dynamic simulations, strawberry

## Abstract

The endo-β-1,4-glucanases (EGs) that belong to the glycosyl hydrolase family 9 (GH9) have roles in cell wall synthesis, remodeling and degradation. Previous studies have suggested that EGs may play a key role in the ripening of different fruits including strawberries. In this study, we used reverse-transcription quantitative polymerase chain reaction (RT-qPCR) assays to determine the transcript accumulation of an endo-β-1,4-glucanase (*FaEG1*) during fruit development in two different strawberry ‘Camarosa’ and ‘Monterey’ with contrasting softening ratios. Phylogenetic analyses suggest that *FaEG1* belongs to the α group of the GH9 family with other proteins previously described with roles in elongation, abscission and ripening. Comparative modeling was used to obtain the FaEG1 structure. The model displays a α-barrel–type structure that is typical of the GH9 enzyme family, and comprises 12 α-helices, 2 3_10_ helices and 6 β-sheets. The catalytic residues were oriented to the solvent in the middle of an open groove. Protein–ligand interactions were explored with cellulose and two xyloglucans as ligands; the results suggest that the FaEG1-cellulose and FaEG1-XXXGXXXG (the most abundant xyloglucan in strawberries) complexes were more stable complexes than XXFGXXFG. The cell wall degradation was observed by scanning electron microscopy (SEM). The data are congruent with the probable role of the FaEG1 protein in the dissembly of the cellulose-hemicellulose fraction during the ripening of strawberry fruit.

## 1. Introduction

Commercial strawberries are among the most widely consumed fruits in the world, nevertheless these fruits exhibit rapid softening during ripening [1,2]. Fruit softening that takes place during ripening has been associated with the degradation or disassembly of the different cell wall components [3]. Recently, Castro and Morales-Quintana [4] performed a comparative study of the changes in the physiological properties and cell wall-associated polysaccharide contents during the different stages of ‘Camarosa’ strawberry ripening [4]. This study was based on the thermogravimetric analysis (TGA) curves. The authors demonstrated that a lower thermal stability exists in the ripe stage sample than that in the green stage (initial ripening stage) [4]. These differences in the TGA curve could result from plant cell wall metabolism. Different authors have described that the disassembly is the main process that leads to fruit softening during ripening and postharvest [1,2,5,6,7]. For this reason, several enzymes and proteins have been widely studied [6,7,8,9,10].

Thus, within the different families of enzymes studied to date, three types of this hydrolytic enzymes that are actively implicated in the cell wall disassembly are the endo-1,4-β-glucanases (EC 3.2.1.4), cellobiohydrolases (EC 3.2.1.91), and β-glucosidases (EC 3.2.1.21). These three enzymes act synergistically to degrade the cellulose polymers [11], cellulose is one of the major components of the plant cell walls [12,13], and in strawberries they correspond to approximately 30% of the plant cell wall. Firstly, the endo-1,4-β-glucanases catalyze the initial attack to initial degradation on the cellulose polymer and account for the random cleavage of the β-1,4-glycosidic bonds present in the cellulose chain [14]. After this, smaller fragments are released, and this facilitates subsequent hydrolysis by the other two cellulases mentioned [15]; for this reason, it is important to know the mode of action at the molecular level of the endoglucanase enzyme.

In plants, the endo-1,4-β-glucanases (EGases) or ‘cellulases’ enzymes have long been studied in processes where the separation of cells is required [14]. The glycosyl hydrolase family 9 (GH9) clustered EGases, and some enzymes members of GH9, are associated with various aspects of plant growth, such as cell wall metabolism, and cellulose biosynthesis [16,17,18]. GH9 members may indirectly affect cell expansion by changing the viscosity and/or porosity of the cell wall. Thus, this protein promotes the activity of other cell wall-loosening factors, such as expansions, as has been suggested previously [19].

To date, genes encoding the GH9 enzymes have been isolated primarily from model plants and economically important crops. In *Arabidopsis thaliana*, the phylogenetic analysis of the *EG* gene family showed three EG subfamilies (α, β, and γ), two of which (α and β) contain secreted EGases involved in a variety of physiological processes (elongation, ripening, and abscission) and one (the γ-subfamily) is composed of proteins predicted to have a membrane-spanning domain and to be localized at the plasmalemma [20,21]. In another model crop in study, such as rice (*Oryza sativa*), the global identification of multiple OsGH9 family members revealed 25 putative GH9s enzymes [22]. Regarding softening during fruit ripening, different EGases have been described at the molecular level, including the fruits of strawberry [23,24,25,26], tomato [17], avocado [27], and pepper [28,29].

Specifically, in strawberry the expression levels of the two *endoglucanase* genes (named as *FaEG1* and *FaEG2*) were studied by RNA gel blot analysis; the two *endoglucanases* are significantly expressed in fruits [25,26], and one of them (*FaEG3*) is expressed in growing vegetative tissues [26,30]. Interestingly, *FaEG1* is fruit-specific, and starts to be expressed at the stage of white fruits, that is when the fruits stop their growth and enter into the ripening phase; therefore, it would seem important to characterize the FaEG1 enzyme, and thus, improve our understanding of the softening process in strawberies. Even though it seems that these proteins have been extensively studied at the biochemical and molecular levels, there are no structural or computational studies of this or another endoglucanase strawberry enzyme.

Despite the knowledge generated in the past few years, there is still a limited understanding of the molecular mechanisms involved in cell wall remodeling. Regarding this lack of information, it is relevant to clarify the interaction of EG proteins with cellulose polymers and the nature of the mechanism of action at the molecular level for a better comprehension of cell wall metabolism during fruit ripening. Therefore, the aim of this work was to evaluate the expression profile in two strawberry cultivars with contrasting softening ratios and to structurally characterize the FaEG1 enzyme.

## 2. Methods

### 2.1. Plant Material

Fruits from *F. x ananassa* ‘Camarosa’ and ‘Monterey’ were classified according to a previous report by Ramos et al. [2] and were named large green fruit (G), white fruit (W), 50% red fruit (50%), and ripe fruit (R). The fruits were obtained and harvested from plants grown in the same commercial orchard in the town of Pelluhue in the Maule Region, Chile (latitude 35°50’00’’ S; longitude 72°38’00’’ W) according to Ramos et al. [2] and Parra-Palma et al. [31]. A total of 30 fruits were collected from each cultivar and each developmental stage.

### 2.2. RNA Isolation, Reverse Transcription and Reverse-Transcription Quantitative Polymerase Chain Reaction (RT-qPCR) Analysis

Total RNA was isolated from frozen fruit using CTAB method similar to Ramos et al. [2]. Contaminant genomic DNA was removed using TURBO DNA-free™ Kit (Ambion, Life Technologies) according to the manufacturer’s procedure. RNA concentration and integrity were determined by Qubit 4 fluorometer (Invitrogen) and agarose gels respectively. A bulk of each development stage was obtained from the mixture of six complete fruits; one independent RNA extraction was carried out from three independent bulks to each fruit developmental stage, obtaining three extractions for each one. First-strand complementary DNA (cDNA) synthesis was performed using a First Strand cDNA Synthesis Kit (Fermentas Life Science, Glen Burnie, MD, USA) following the manufacturer’s instructions.

The mRNA abundance of the *FaEG1* gene was measured by reverse-transcription quantitative polymerase chain reaction (RT-qPCR) analysis. Reaction and quantification were performed following the procedure described by Ramos et al. [2] and Parra-Palma et al. [31]. Forward and reverse primers used for RT-qPCR analysis are 5’-CCACGGGCTCTATCAAAATC-3’ and 5’-TGGCCTTCGAAGAAGAGG-3’, respectively. Each reaction were performed in triplicate and normalized against the expression level of *F. x ananassa glyceraldehyde-3-phosphate-dehydrogenase 1* (*FaGAPDH1*) gene obtained from [2], additionally a negative water control was included. Fluorescence was measured at the end of each extension step.

### 2.3. Scanning Electron Microscopy (SEM)

The scanning electron microscopy (SEM) method was used to examine morphological changes in the cell wall of the different strawberry stages. Specifications—accelerating voltage: 15 KV; and secondary electron image; working distance: 15 mm all the samples were not conductive; they were coated with 10 mm of gold (sputtering technique) and were examined with SEM (JEOL JSM-6380LV, Japan).

### 2.4. Sequence Comparison and Phylogenic Analysis

The FaEG1 protein sequence from strawberries was retrieved from the NCBI with the GenBank accession number: AJ414709. The protein sequences were aligned with 25 other sequences of the *Arabidopsis endo-1,4-glucanase* gene family [20] using clustalW software [32] with European Molecular Biology Laboratory - European Bioinformatics Institute (EMBL-EBI) used as the default parameter. MEGA software version 6.06-mac [33,34,35] was used for tree development and phylogenic analysis. The phylogenic tree was built using the neighbor-joining and bootstrap analysis method (5000 replicates).

### 2.5. FaEG1 Structural Model Obtained by Comparative Modeling

The comparative model of FaEG1 was generated according to the method employed by Morales-Quintana et al. [36] and using MODELLER 9v17 software (http://salilab.org/modeller/) [37]. Crystal structure of endo-1,4-β-glucanase from termite (*Nasutitermes takasagoensis*) (Protein Data Bank (PDB) code: 1KS8) was selected as template based on the sequence identity. Desmond Package of SCHRÖDINGER suite with the OPLS v2005 force field [38] was used in a molecular dynamics (MD) simulation of 10 ns to refine the FaEG1 structure and equilibrate the system with a SPC water model. To calculate long-range electrostatic interactions we used the particle mesh Ewald (PME). The PROPKA program and SCHRÖDINGER suite were used to set the protein protonation state in pH 5.5 according to Wang et al. (2008) [39], who described it as the pH at which the EGs enzymes have the highest activity. To evaluate the quality of the different models generated, both PROCHECK [40] and TM-score [41] were performed to evaluate the similarity of folding between the FaEG1 protein models and the template structure. Additionally, the ProSA-Web [42,43] program was employed to evaluate the energies of the structural models.

### 2.6. Protein–Ligand Interaction Determinations

Molecular docking studies were performed using Autodock Vina program v1.1.2 [44] to predict the putative binding interaction modes of the FaEG1 with three different octasaccharides (cellulose molecule according to [45] and two different xyloglucans (XG) (XXXGXXXG and XXFGXXFG polymers). The methodology was realized according to [6,46]. After the molecular docking, each ligand was evaluated inside to the active site of FaEG1 structural model by molecular dynamics (MD) simulation. The protein model was embedded in the pre-equilibrated SPC model of water molecules, adding NaCl neutralized the system. All MD simulations were performed at constant temperature (300 K) and pressure (1.01325 bar). The MD simulation was run over 200 ns, and the motion equations were integrated with 2 fs, while the data were collected for every 100 ps of trajectory. The Desmond Package of SCHRÖDINGER suite with the OPLS v2005 force field [38] was used in the MD simulations. Finally, the MD simulations were analyzed using VMD software [47].

## 3. Results and Discussion

### 3.1. Sequence and Phylogenetic Analyses

**Figure 1:**
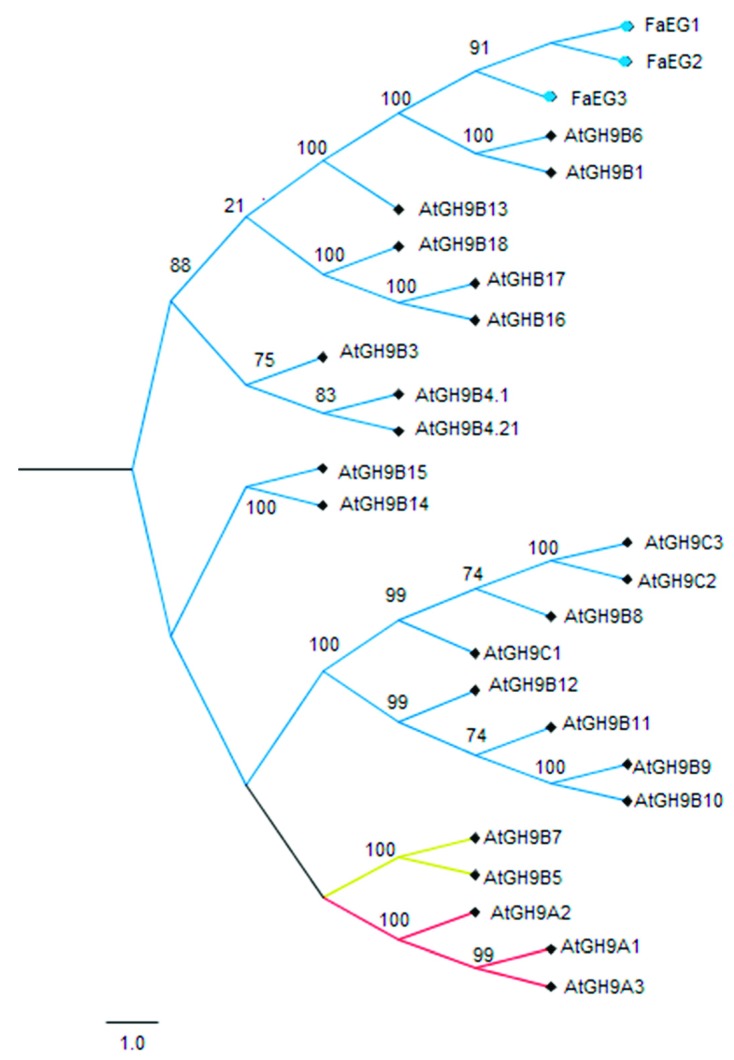
Phylogenic analysis of *FaEG1* with different orthologs sequences from *Arabidopsis thaliana* and *F. x ananassa*. The phylogenetic tree was constructed using the neighbor-joining method (Jones–Taylor–Thornton substitution model) and bootstrap analysis of 5000 replicates.

FaEG1 sequence analysis showed that the deduced protein has a molecular weight of 54.9 kDa, with an isoelectric point of the protein is 9.18 including the signal peptide. Meanwhile, the mature protein has a molecular weight of 51.4 kDa and an isoelectric point of 9.0. In these terms, a high probability extracellular localization was deduced by analyzing the signal peptide, with a length of 33 amino acids. With respect to the identity, the FaEG1, FaEG2 and FaEG3 amino acid sequences were aligned and compared with the other 25 *Arabidopsis* endoglucanases (Figure S1), the FaEG1 amino acid sequence showed a high identity level with the other two strawberry sequences (99% of sequence identity with each one) (Figure S2). With respect to the *Arabidopsis* comparison, the FaEG1 amino acid sequences were 79.8% and 76.4% identical to the AtGH9B1 and AtGH9B6 sequences, respectively (Table S1), while FaEG2 showed 79.6% and 76.2% identity to the AtGH9B1 and AtGH9B6 sequences, respectively, and FaEG3 showed respective 79.2% and 75.8% identities with the same sequences. A phylogenic tree was built from the previous multiple sequence alignment, and the grouping pattern provides three main subgroups (Figure 1). As expected, the three strawberry sequences were grouped together. The FaEG1 amino acid sequence was clustered into the α subgroup with other EG enzymes related to the ripening process (Figure 1). Members of the α subfamily of the endoglucanases have been described for their great importance in the ripening process [20,21]. Thus, these results would indicate that FaEG1 is involved in the process of fruit ripening and are in accordance with those suggested by Trainotti et al. [26].

### 3.2. Transcriptional Profile Analysis of the *FaEG1* Gene

Strawberry fruit softens quickly during development and ripening [1]. We recently observed that the main fruit firmness reduction occurred between the large green (G) and white (W) stages in the same four different strawberry cultivars [2]. Change firmness reduction in fruit softening or loss of firmness was near 80% from G to ripe (R) stages in ‘Camarosa’, with ‘Camarosa’ being the cultivar that showed greater firmness, or it was the cultivar that showed the smallest reduction; in contrast, ‘Monterey’ showed a reduction near to 90% from the G to R stages, showing a greater firmness loss [2].

**Figure 2:**
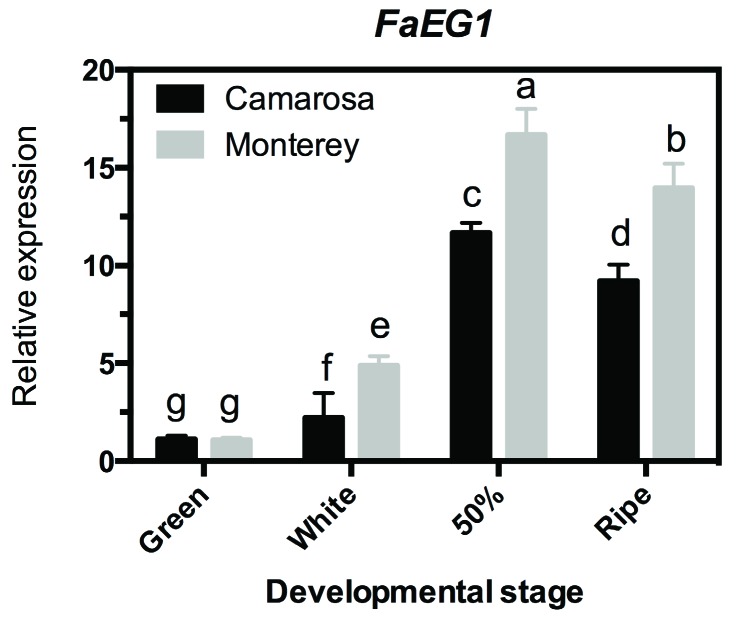
Changes in *FaEG1* mRNA levels measured by reverse-transcription quantitative polymerase chain reaction (RT-qPCR) during fruit development and ripening of the different strawberry (*Fragaria x ananassa*) cultivars. The expression data of the *FaEG1* correspond to the mean of three replicates normalized against *FaGADPH1* abundance. Different letters indicate significant differences between cultivars and stages (*P* < 0.05).

To evaluate the expression profile of the *FaEG1* gene in the two strawberry ‘Camarosa’ and ‘Monterey’ with contrasting softening levels, an RT-qPCR transcriptional analysis was performed in whole fruit samples from four different developmental stages per cultivar (Figure 2). The transcript abundance was higher in ‘Monterey’ than that in ‘Camarosa’ cultivars in three of the four stages evaluated; this significant difference was observed in ‘Monterey’ at the W, 50% and R stages, with a peak of abundance at the 50% stage (Figure 2). This is in accordance with results described previously, where ‘Monterey’, exhibits the lower fruit firmness at the ripe stage [2]. Additionally, these results are similar to those described by [26] using an RNA gel blot analysis, which showed an increase in the transcript accumulation level during the ripening process of ‘Chandler’ strawberry fruit [26]. Thus, it would show the confirmation that this gene would be involved in the process of fruit ripening.

Along these lines, different authors showed that the ripening-associated softening of fleshy fruit is mainly a consequence of enzyme-mediated cell wall disassembly [3], and the endo-1,4-β-glucanases would be an important molecular component in this process. The increase in the mRNA levels of FaEG1 supports this hypothesis. In strawberries, the textural changes were related to the loosening of the xyloglucan-cellulose network [2,6,7] and were related to the depolymerization and solubilization of hemicelluloses and pectins within the cell wall, respectively [1,2]. Recently, the integrity, structural organization and stability of the polymers that conform to the cell wall in the fruit during the ripening process were evaluated with TGA curves [4]. The different ripening stages of ‘Camarosa’ fruits were used to show that the green stage (the initial ripening stage evaluated) had the greatest thermal stability, while the ripe stage sample had less thermal stability [4]. These differences in thermal stability possibly are the result of the action of various hydrolytic enzymes, including polygalacturonase, pectate lyase, xyloses, expansins, and xyloglucan endotransglucosylase/hydrolases. These are in addition to the 1,4-β-glucanases that produce polysaccharide solubilization, degradation and depolymerization. Now, if the above is true, the structure of the cell wall should change throughout the development and ripening of the strawberry fruit, and this could be reflected with microscopy studies.

### 3.3. SEM Studies of the Surface Morphology of Strawberries

**Figure 3:**
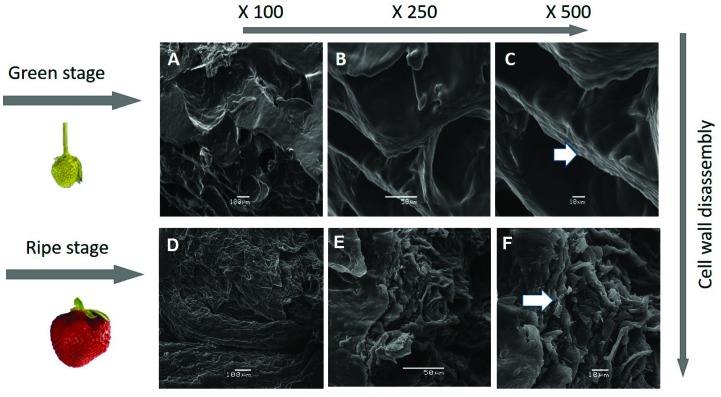
Scanning electron micrographs (SEM) of *F. x ananassa* ‘Camarosa’. (**A**–**C**): Green stage; (**D**–**F**): Ripe stage. The white arrow showed the disassembly of the cellulose-hemicellulose fraction.

Changes in fruit texture taking place during ripening, described as softening, are mainly due to alterations in the structure and/or composition of the cell wall. To show this effect in the surface morphology of the two contrasting ripening stages of *F. x ananassa,* ‘Camarosa’ was studied, as a representative case, through SEM (Figure 3). The cell wall disassemble is observed in the Figure 3 and is an important factor in determining the firmness or resistance of the cell wall of the strawberry during the different ripening stages. The analysis is revealed the complex ordered structure made of hemicellulose and cellulose (Figure 3A–C). Additionally, the change in the surface morphology due to the disassembly of the polymeric matrix fruit and ripening is associated with a decrease in firmness previously described in ‘Camarosa’ and other cultivars [1,2]. In contrast, an amorphous system is observed in Figure 3D–F as result of depolymerization or degradation and cell wall degradation; low molecular weight compounds are then generated from the cell wall polymer components (Figure 3).

### 3.4. Obtaining the FaEG1 Protein Model

**Figure 4:**
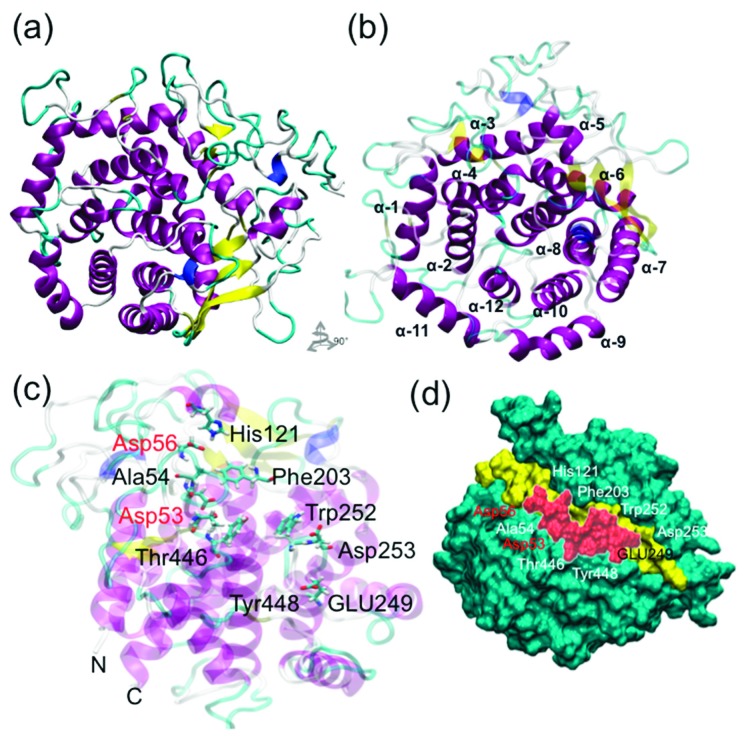
FaEG1 protein model. (**a**) Frontal view of the FaEG1 3D-structure that was built using the 1KS8 protein as a template (40.5% identity). (**b**) Top view of the FaEG1 structural model. (**c**) Important residues involved in the protein-ligand interaction, the oriented in the open groove is showed, in red the two catalytic residues. (**d**) Surface of the FaEG1 protein model shown in green, while the open groove is shown in yellow color.

To determine the structural properties of the FaEG1 enzyme, a protein model was built using the information provided by the sequence alignment between the template and the FaEG1 enzyme (Figure S3). The identity of the amino acid sequence was 40.5% between both sequences. The structural model obtained for the FaEG1 showed a α-barrel-type folding (Figure 4a). This folding pattern is common in proteins of the glycosyl hydrolase family 9 (GH9) [16,17,18]. Thus, 6 β-sheets, 12 α-helices and 2 3_10_-helices would form the structure of the FaEG1. The 12 helices have an alternating connectining design (Figure 4b), with a variation in the inner and outer helices and internal helices that are in the parallel form of a central cylinder (Figure 4b). Additionally, at the C-terminal these helices are connected by short loops.

The binding site showed the structure of the open groove (Figure 4c,d), which is characteristic of these enzymes, and the binding site is oriented towards the amino-terminal of the enzyme and is formed by 6 internal helices (Figure 4c). The active site of the FaEG1 corresponds to two aspartate residues [16], and in FaEG1 corresponds to the Asp53 and Asp56 residues. The residues Asp53 and Aps56 were oriented toward the solvent in the middle of the open groove. Additionally, other important residues described in the GH9 family are Tyr448, His121 and Trp124 (using number of FaEG1 as reference) [16,17,18], and similar to the two catalytic residues, these three residues were oriented toward the solvent near to the open groove (Figure 4c).

### 3.5. 3D Structure of the FaEG1 Protein Model

Firstly, the Root Mean Square Deviation (RMSD) value of the backbone calculated between the FaEG1 and the structural template was 2.59 Å, and the model showed a similar secondary structure; however, in the loops region present in FaEG1 there were differences with respect to the template. Using the PROCHECK program we evaluated the stereochemical quality of the model. The analysis showed that 100% of the amino acid residues were classified in any of the three favored regions (most favorable region, additionally allowed region, generously allowed region) (Table S2). With respect to the TM-score analysis, the result showed that FaEG1 has the same fold as that of the template with a score of 0.6801. In energetic terms, the global z-score of FaEG1 was −7.32, and the template score was −8.66, which was obtained from the ProSA analysis. Both values were within the accepted range for proteins of this size according to the ProSA parameters [43]. This form, the final structural model of FaEG1 (Figure 4a), was accepted for subsequent analysis.

### 3.6. Protein–Ligand Interactions

**Figure 5:**
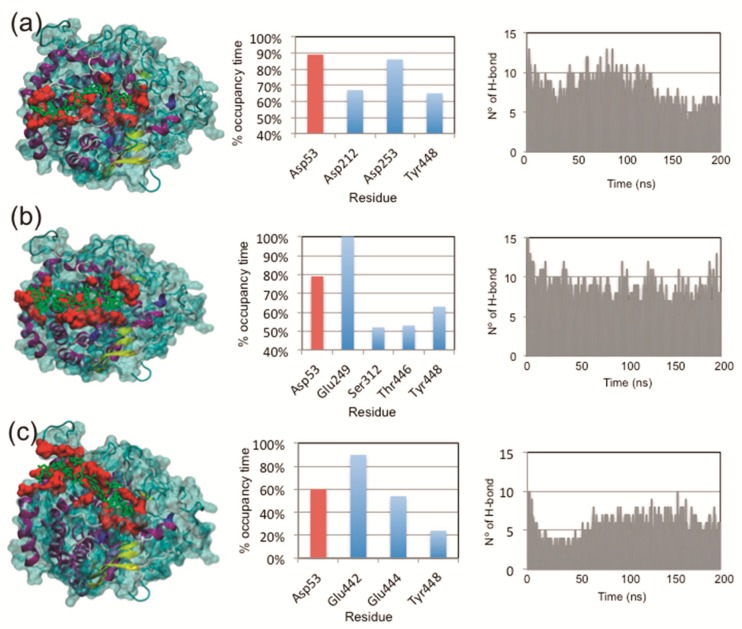
MDS analyses of the interaction of FaEG1 with cellulose (**a**) and two hemicellulose octasaccharides: XXXGXXXG (**b**) and XXFGXXFG (**c**). In the left, a general view of the interaction of the protein and the corresponding ligand. In the center, a graph indicating the percentage of time that a hydrogen bond between particular amino acid residues and the ligand is established; the catalytic residues are shown in red. On the right are the number of hydrogen bonds established during MDS between the ligand and different amino acid residues located in the open groove of each protein model. Only values greater than or equal to 40% frequency are shown in the center graph to (**a**) and (**b**) graphics, while the values greater than or equal to 10% frequency are shown in the (**c**) center graph.

A protein-ligand interaction study was carried out using two approximations: molecular docking and molecular dynamic simulations. Firstly, the protein–ligand conformation was evaluated using an automatic docking analysis, and as shown in Table 1, favorable binding energies were obtained for the protein interacting with the three ligands evaluated. Interestingly, no significant differences were found in the interaction of FaEG1 with the cellulose and XXXGXXXG or XXXGXXXG and XXFGXXFG, while significant differences were found in cellulose with respect to XXFGXXFG (Table 1). Secondly, using MD simulations, with the aim of explaining the differences in binding energy observed in docking studies, the interactions associated with the protein–ligand complexes were studied. The Figure 5 (left panel) shows the orientation of the ligands in the FaEG1 model obtained from the different MD simulation analyses. In this figure, the three ligands were generally oriented in the open groove (Figure 5, left panel). However, it can be observed that the cellulose and XXXGXXXG are positioned along the open groove of the protein, while XXFGXXFG was oriented slightly over the open groove (Figure 5a–c, left panel).

With respect to the residues within the open groove that interact with each ligand, the three ligands interact with the catalytic residue (Asp53) with occupancies over 50% during the MD trajectory (Figure 4a–c, central panel, red bars). However, the protein–ligand complex with cellulose showed the greatest occupancy time (Figure 5, central panel). Strangely, the other catalytic residue (Asp56) does showed occupancy times of 32.6%, 18.9% and 13.1% for cellulose, XXXGXXXG and XXFGXXFG, respectively. Those values were found to be lower than those of other less important residues in catalytic terms. On the other hand, during the first 100 ns of the MD simulation of the two of the complexes, no differences were found in the number of hydrogen bonds (H-bonds) formed between cellulose or XXXGXXXG with the FaEG1 protein model, and both MD simulations showed the formation of approximately 10 H-bonds. After this time, the number of H-bonds in the MD simulation of the FaEG1-cellulose complex began to decrease, showing approximately seven H-bonds, while for the other complex with the XXFGXXFG ligand, this value oscillated more with values approximately seven or 10 (Figure 5a,b, right panel). With respect to the XXFGXXFG ligand, significant differences were observed in this complex with respect to the other two, showing only 6 or 7 H-bonds during all MD simulations (Figure 5c, right panel).

Molecular Mechanics/Generalized Born Surface Area (MM-GBSA) calculations for each protein-ligand complex were performed to obtain more reliable estimations of free binding energy (Table 2). The analysis agrees with the results of the docking experiments, as the affinity for cellulose and XXXGXXXG are considerably lower than that for XXFGXXFG (Table 2). The total binding energy indicates that the complex between FaEG1 and cellulose is the most stable, followed by XXXGXXXG (Table 2).

Finally, the RMSD values of each protein-ligand complex were tested to check the convergence of the calculations and MD trajectory during the simulation. The RMSD values remained at approximately 1–1.5 Å for the FaEG1-XXXGXXXG and FaEG1-cellulose systems, while the FaEG1-XXFGXXFG complex showed an RMSD value of approximately 2.5 Å during the first 80 ns. After that time, the RMSD value dropped to levels similar to those observed in the FaEG1-cellulose and FaEG1-XXXGXXXG systems, demonstrating the conformational stability of the different protein–ligand structures (Figure S4).

## 4. Conclusions

Our study provides useful information on the FaEG1 structural model and predicts a better binding interaction with cellulose and XXXGXXXG over other hemicelluloses, such as XXFGXXFG. Overall, the data provided allow us to propose that FaEG1 might be involved in the disassembly of the cellulose and hemicellulose framework and we speculate that the FaEG1 enzyme would plays a role in cell wall remodeling during the rapid softening of *F. ananassa* fruits and tissues that requires further experiments in other cell wall-degrading enzymes.

## Figures and Tables

**Table 1:** Affinity energies for the different protein–ligand complexes.

Complex		Binding Energy (Autodock Vina Score)
FcEG1	cellodextrin 8-mer	−7.5 ^a^ ± 0.4
	XXXGXXXG	−6.9 ^ab^ ± 0.5
	XXFGXXFG	−5.9 ^b^ ± 0.5

Different superscripted letters indicate significant differences of FaEG1 protein with the different ligands. (Tukey Honestly-significant-difference (HSD) test, *P* = 0.05).

**Table 2:** MM-GBSA analysis for the interaction of the different complexes formed between FaEG1 with the different XG and cellulose ligands.

Complex		ΔH^vdW^_MM_ (kcal mol^−1^)	ΔH^elec^_MM_ (kcal mol^−1^)	ΔG_sol–pol_ (kcal mol^−1^)	ΔG_sol–npol_ (kcal mol^−1^)	ΔG_bind_ (kcal mol^−1^)
FcEG1	Cellulose	−67.21	−71.11	67.30	−52.30	−123.32 ± 9 ^a^
	XXXGXXXG	−55.21	−58.14	63.10	−25.06	−75.31 ± 5 ^ab^
	XXFGXXFG	−52.85	−26.51	46.68	−30.97	−63.65 ± 8 ^b^

ΔH^vdW^_MM_, van der Waals contributions; ΔH^elec^_MM_, electrostatic contribution; ΔG_sol–pol_, ΔG_sol–npol_, contribution of solvation; ΔG_bind_, total binding energy. Different superscripted letters indicate significant differences in each protein with the different ligands. (Tukey HSD test, *P* = 0.05).

## Supplementary Materials

The following are available online at https://www.mdpi.com/2223-7747/8/6/140/s1: Figure S1: Comparison of FaEG1 sequence from *Fragaria x ananassa* with their orthologs, Figure S2: Comparison of FaEG1 sequence from *Fragaria x ananassa* with the 1KS8 sequence used as template, Figure S3: RMSD of the protein ligand complex, Table S1: Sequence Identity matrix, Table S2: Validation of FaEG1 protein using the PROCHECK, ProSA, and Verify3D programs.
