# Supplementary material for: Molecular Insights into FaEG1, a Strawberry Endoglucanase Enzyme Expressed during Strawberry Fruit Ripening
Source: Plants (Basel). 2019 May 28;8(6):140. doi: 10.3390/plants8060140 (PMC6631567; doi:10.3390/plants8060140)
Supplement: Supplementary file 1 [file plants-08-00140-s001.zip › plants-501458-suppl-final/Table S2.docx]

**Table S2. Validation of FaEG1 protein structure using the PROCHECK, ProSA and Verify3D programs.**

| **Structure** | **PROCHECK** | | | | **ProSA** | **Verify3D** |
| --- | --- | --- | --- | --- | --- | --- |
|  | **Most favorable region (%)** | **Additional allowed regions (%)** | **Generously allowed regions (%)** | **Disallowed regions (%)** | **Z-score** | **Residues (%) with score over 0.2** |
| FaEG1 | 89.9 | 9.3 | 0.8 | 0.0 | -7.32 | 94.9 |
