# Supplementary material for: Molecular Insights into FaEG1, a Strawberry Endoglucanase Enzyme Expressed during Strawberry Fruit Ripening
Source: Plants (Basel). 2019 May 28;8(6):140. doi: 10.3390/plants8060140 (PMC6631567; doi:10.3390/plants8060140)
Supplement: Supplementary file 1 [file plants-08-00140-s001.zip › plants-501458-suppl-final/Figures Supplementaries.doc]

```
                     10         20         30         40         50         60         70         80         90        100        110        120
            ....|....| ....|....| ....|....| ....|....| ....|....| ....|....| ....|....| ....|....| ....|....| ....|....| ....|....| ....|....|
AtGH9B9     ---------- ---------- ---------- ---------- ---------- ---------- ----MSQSKN GSSQCLWTSI CIVVLVMSMA RGAVS----- ---------- ----TNYAEA
AtGH9B10    ---------- ---------- ---------- ---------- ---------- ---------- ----MSQLKN GSSQCLWTSI CIVLIVMSMA REAVS----- ---------- ----TNYAEA
AtGH9B11    ---------- ---------- ---------- ---------- ---------- ---------- ----MSQLKI GSSQCLWTSI CIVLFVLSMA RGAVS----- ---------- ----RNYGDA
AtGH9B12    ---------- ---------- ---------- ---------- ---------- ---------- ----MSCISS QCFITIKSIC IVLLLSITCG AVSAN----- ---------- ----SNYGEA
AtGH9C3     ---------- ---------- ---------- ---------- ---------- ---------- ---------M GSRTTISILV VLLLGLVQLA IS-------- ---------- ---GHDYKQA
AtGH9C2     ---------- ---------- ---------- ---------- ---------- ---------- ---------- MEKFAPVAAL LLLLLCFPVA FS-------- ---------- ---GHDYGQA
AtGH9B8     ---------- ---------- ---------- ---------- ---------- ---------- -----MTVMN HRHKQCHFLL LLLITVFSAA LDGVSSETDV ---------- --GGFDYGEA
AtGH9C1     ---------- ---------- ---------- ---------- ---------- ---------- -----MRKFG GSLFGVSLLL SVLLAAATAA AEY------- ---------- ----YNYGSA
AtGH9B6     ---------- ---------- ---------- ---------- ---------- ---------- ---------- MAGKSFMTPA IMLAMLLLIS PETYA----- ---------- ---GHDYRDA
ATGH9B1     ---------- ---------- ---------- ---------- ---------- ---------- ---------- MARKSLIFPV ILLAVLLFSP PIYSA----- ---------- ---GHDYRDA
FcEG1       ---------- ---------- ---------- ---------- ---------- ---------- ---MARNGLC LPGNAPAFRA TLVLSLLLLL QPIRA----- ---------- ---GHDYHDA
AtGH9B13    ---------- ---------- ---------- ---------- ------MALL LVSSSSSYAL RVTIFLSFFF FLCNGFSYPT TSSLFNTHHH RHHLA----- ---------- ---KHNYKDA
ATGH9B4.1   ---------- ---------- ---------- ---------- ---------- ---------- ---------- ----MASPFF FVFLLSALSL ENTYA----- ---------- ---SPNYREA
ATGH9B4.2l  ---------- ---------- ---------- ---------- ---------- ---------- ---------- ---------- ---------- ---------- ---------- ----------
ATGH9B3     ---------- ---------- ---------- ---------- ---------- ---------- ---------- ----MTSLFF FVLLFSSLLI SNGDA----- ---------- ---NPNYKEA
AtGH9B17    ---------- ---------- ---------- ---------- ---------- ---------- ---------- ---MKASIYL VTVFILLLLL LPTAI----- ---------- ---PHDYSDA
AtGH9B16    ---------- ---------- ---------- ---------- ---------- ---------- ---------- -----MKPLV CSFIVILLIL LPTTI----- ---------- ---SHDYSDA
AtGH9B18    ---------- ---------- ---------- ---------- ---------- ---------- ---------M KLHLHPLSLV FFFFFFFFRP TMSSN----- ---------- ---QHDYSDA
AtGH9B15    ---------- ---------- ---------- ---------- ---------- ---------- ---------- -----MGKLL VLMLVGMFLA FESLE----- ---------- ---ALEYGDA
AtGH9B14    ---------- ---------- ---------- ---------- ---------- ---------- ---------- -----MGKLL VVMLIGMFLA FESLE----- ---------- ---ALDYGDA
AtGH9B7     ---------- ---------- ---------- ---------- ---------- ---------M EEKSKSRGWC GWFIAIIVLA SVILAVVYTV KLRTKKSGDD DGGGPVPGPP GAIDKKYADA
AtGH9B5     ---------- ---------- ---------- ---------- ---------- --------MV AKPRSRCCCC SVFIGVIILI AIIIAVIFTI RHRSNHSDDD ---------- GSNVKNYANA
ATGH9A1     MYGRDPWGGP LEINTADSAT DDD--RSRNL NDLDRAALSR ---PLDETQQ SWLLGPTE-- -QKKKKYVDL GCIIVSRKIF VWTVGTLVAA ALLAGFITLI VKTVPRHHPK TPPPDNYTIA
ATGH9A3     MYGRDPWGGP LEINAADSMT DDD--RSRNL QDLDRATPSR ---PLDETQQ SWLLGPKV-- -LKKKKYVDL GCILVSRKIF LWTLGTIVVT ALLSGFITLI VKTLPHHHHK EPPPDNYTIA
ATGH9A2     MHPGNVWGGS LDAVDSDRIA AEEEERLRNT TEWDRGAIHS QRSELDETQQ GWLLAPQDNW RKKKKKYVNL GCVSVSRTVF LWTVGSIAVL FLVVALPIII VKSLPRHKSA PPPPDNYTLA


                    130        140        150        160        170        180        190        200        210        220        230        240
            ....|....| ....|....| ....|....| ....|....| ....|....| ....|....| ....|....| ....|....| ....|....| ....|....| ....|....| ....|....|
AtGH9B9     LQKSLLYFEA QRSGKLPPNQ K-VTWRGDSA LKDG----SD AHIDLTGGYY DAGDNMKFGF PLAFTTTMLA WSNIEMGSEL RAHHEQGNSL RALKWATDYL IKAH----PQ PNVLYGQVGE
AtGH9B10    LKNSLLYFEA QRSGKLPPNQ R-VTWRGDSA LRDG----SD AHIDLTGGYY DAGDNMKFGF PLAFTTTMLA WSNIEMASQL RAHHEKGNAL RALKWATDYL IKAH----PQ PNVLYGQVGE
AtGH9B11    LTKSLLYFEA QRSGKLPSNQ R-VTWRGDSA LRDG----SD AHVDLTGGYY DAGDNMKFGF PLAFFTTMLA WSNIEMATQL KAHQEQENAL AALKWATDFL IKAH----PE PNVLYGQVGD
AtGH9B12    LTKSLLYFEA QRSGKLPLDQ R-VIWRGDSA LRDG----SD AHVDLTGGYY DAGDNMKFGF PLAFTTTMLA WSSVEMESQL KAHQEHGNTL AALRWATDYL IKAH----PE PNVLYGQVGD
AtGH9C3     LSKSILFFEA QRSGHLPPNQ R-VSWRSHSG LYDG----KS SGVDLVGGYY DAGDNVKFGL PMAFTVTTMC WSIIEYGGQL ESNGELGHAI DAVKWGTDYF IKAH----PE PNVLYGEVGD
AtGH9C2     LSKSLLFFEA QRSGVLPRNQ R-VTWRSHSG LTDG----KS SGVNLVGGYY DAGDNVKFGL PMAFTVTMMA WSVIEYGNQL QANGELGNSI DAIKWGTDYF IKAH----PE PNVLYGEVGD
AtGH9B8     LSKSLLYFEA QRSGRLPYNQ R-VTWRDHSG LTDG----LE QGVDLVGGYH DAGDHVKFGL PMAFTVTMLS WSVIEYGDSL ASTGELSHAL EAIKWGTDYF IKAH----TS PNVLWAEVGD
AtGH9C1     LDKTFLFFEA QRSGKLPAAQ R-VKWRGPSG LKDG----LA QGVSLEGGYY DAGDHVKFGL PMAFAVTMLS WAAVDNRKEL SSSNQMQQTL WSIRWGTDYF IKAH----PQ PNVLWGQVGD
AtGH9B6     LRKSILFFEG QRSGKLPPDQ R-LKWRRDSA LRDG----SS AGVDLTGGYY DAGDNVKFGF PMAFTTTMMS WSVIDFGKTM G-PELENAVK AIKWGTDYLM KAT-----QI PDVVFVQVGD
ATGH9B1     LRKSILFFEG QRSGKLPPDQ R-LKWRRDSA LRDG----SS AGVDLSGGYY DAGDNIKFGF PMAFTTTMLS WSIIDFGKTM G-PELRNAVK AVKWGTDYLL KAT-----AI PGVVFVQVGD
FcEG1       LRKSILFFEG QRSGKLPPDQ R-LKWRRDSA LHDG----ST AGVDLTGGYY DAGDNVKFGF PMAFTTTLLA WSIIDFGRVM G-TEQRNAVK ALRWGTDYLL KAT-----AV PGVVFVQVGD
AtGH9B13    LTKSILFFEG QRSGKLPSNQ R-MSWRRDSG LSDG----SA LHVDLVGGYY DAGDNIKFGF PMAFTTTMLS WSVIEFGGLM K-SELQNAKI AIRWATDYLL KAT-----SQ PDTIYVQVGD
ATGH9B4.1   LSKSLLFFQG QRSGRLPSDQ Q-LSWRSSSG LSDG----SS AHVDLTGGYY DAGDNVKFNF PMAFTTTMLS WSSLEYGKKM G-PELQNSRV AIRWATDYLL KCAR----AT PGKLYVGVGD
ATGH9B4.2l  ---------- ---------- ---------- ---------- ---------- ---------- -MAFTTTMLS WSSLEYGKKM G-PELQNSRV AIRWATDYLL KCAR----AT PGKLYVGVGD
ATGH9B3     LSKSLLFFQG QRSGPLPRGQ Q-ISWRASSG LSDG----SA AHVDLTGGYY DAGDNVKFNL PMAFTTTMLS WSALEYGKRM G-PELENARV NIRWATDYLL KCAR----AT PGKLYVGVGD
AtGH9B17    LRKSILFFEG QRSGRLPKQQ R-MAWRRNSA LNDG----KN LKTDLVGGYY DAGDNVKFHF PMAFTATMLA WSSVDFGRYM SQHDFRHNLV AVKWATDYLL KTVS----QL PNRIFVHVGE
AtGH9B16    LTKSILFFEG QRSGYLPREQ R-MTWRHNSA LNDG----KN LNVDLVGGYY DAGDNIKFHF PMAFTTTMLA WSAIDFGSYM SPADLRDNLV ALRWGSNYLL KTVS----QL PNRIFVQVGE
AtGH9B18    LSKSILFFEG QRSGYLPNDQ R-MTWRRNSG LSDG----WT HNIDLTGGYY DAGDNVKFNF PMAFTTTMLA WSVIEFGEFM PSSELRNSLV ALRWSSNYLL KSVS----QL PNRIFVQVGD
AtGH9B15    LNKSILFFEG QRSGKLPTNQ R-VKWRADSA LSDG----SL ANVNLIGGYY DAGDNVKFVW PMSFTTTLLS WAAIEYQNEI SSVNQLGYLR STIKWGTDFI LRAH----TS PNMLYTQVGD
AtGH9B14    LNKSILFFEG QRSGKLPTNQ R-VKWRADSG LSDG----AS ANVNLIGGYY DAGDNVKFVW PMSFTTTLLS WAALEYQNEI TFVNQLGYLR STIKWGTNFI LRAH----TS TNMLYTQVGD
AtGH9B7     LKLALQFFDI QKSGKLE-NN K-IPWRGDSG LKDG----SE DNLDLSKGLY DAGDHIKFGF PMAFTATVLS WSILEYGDQM NAVNQLDPAK DSLRWITDYL IKAH----PS DNVLYIQVGD
AtGH9B5     LKIAMQFFDI QKSGKLE-NN E-ISWRGDSG LKDG----SE ASIDLSKGLY DAGDHMKFGF PMAFTATVLS WSILEYGDQM ASLNLLDHAK DSLKWTTDFL INAH----PS PNVLYIQVGD
ATGH9A1     LHKALKFFNA QKSGKLPKHN N-VSWRGNSG LQDGKGETGS FYKDLVGGYY DAGDAIKFNF PMAYAMTMLS WSVIEYSAKY EAAGELTHVK ELIKWGTDYF LKTFNSTADS IDDLVSQVGS
ATGH9A3     LRTALKFFNA QQSGKLPKNI YNVSWRHDSC LQDGKGDPGQ CYKDLVGGYY DAGDSIKFNF PMSYAMTMLS WSVIEYSAKY QAAGELEHVK ELIKWGTDYF LKTFNSSADN IYVMVEQVGS
ATGH9A2     LHKALQFFDA QKSGKLPKKN K-VSWRGDSG TKDG---LPD VVGGLVGGYY DGGSNVKFHF PMAFSMTMLS WSLIEYSHKY KAIDEYDHMR DVLKWGTDYL LLTFNNSATR LDHIYTQVGG

                    250        260        270        280        290        300        310        320        330        340        350        360
            ....|....| ....|....| ....|....| ....|....| ....|....| ....|....| ....|....| ....|....| ....|....| ....|....| ....|....| ....|....|
AtGH9B9     G--------N SDHKCWMRPE DMTTPRTSYR IDAQHPGSDL AGETAAAMAA ASIAFAPFDK AYANILIGHA KDLFAFAKAH PGLYQNS--- ITNAGGFYAS SG-YEDELLW AAAWLHRATN
AtGH9B10    G--------N SDHKCWMRPE DMTTPRTSYR IDAQHPGSDL AGETAAAMAA ASIAFAPSDK AYANILIGHA KDLFAFAKAH RGLYQNS--- IPNAGGFYAS SG-YEDELLW AAAWLHRATN
AtGH9B11    G--------N SDHECWMRPE DMTTPRPSFR IDAQHPGSDL AGETAAAMAA ASIAFAPSDE AYAQILIGHA KELFEFAKAY PGIYQNS--- ITNAGGFYAS SG-YEDELLW AAAWLHRATN
AtGH9B12    A--------N LDHACWMRPE DMTTPRPSYR IDPQHPGADL AGETAAAMAA ASLAFAPSDS AYAKTLISHA KELFEFAKDY PGVYHNS--- IPNAGGFYPS SG-YEDELLW AAAWLHRATG
AtGH9C3     G--------K SDHYCWQRPE EMTTDRRAYK IDRNNPGSDL AGETAAAMAA ASIVFRRSDP SYSAELLRHA HQLFEFADKY RGKYDSS--- ITVAQKYYRS VSGYNDELLW AAAWLYQATN
AtGH9C2     G--------N TDHYCWQRPE EMTTDRKAYR IDPSNPGSDL AGETAAAMAA ASIVFRRSNP VYSRLLLTHA YQLFDFADKY RGKYDSS--- ITVAQKYYRS VSGYNDELLW AAAWLYQASN
AtGH9B8     G--------D TDHYCWQRPE DMTTSRRAFK IDENNPGSDI AGETAAAMAA ASIVFRSTNP HYSHLLLHHA QQLFEFGDKY RGKYDES--- LKVVKSYYAS VSGYMDELLW GATWLYRATD
AtGH9C1     G--------E SDHYCWERPE DMTTSRTAYK LDPYHPGSDL AGETAAALAA ASLAFKPFNS SYSALLLSHA KELFSFADKY RGLYTNS--- IPNAKAFYMS SG-YSDELLW AAAWLHRATG
AtGH9B6     A--------Y SDHNCWERPE DMDTLRTVYK IDKDHSGSEV AGETAAALAA ASIVFEKRDP VYSKMLLDRA TRVFAFAQKY RGAYSDS--L YQAVCPFYCD FNGYEDELLW GAAWLHKASK
ATGH9B1     A--------Y SDHNCWERPE DMDTLRTVYK IDRAHPGSDV AGETAAALAA ASIVFRKRDP AYSRLLLDRA TRVFAFANRY RGAYSNS--L YHAVCPFYCD FNGYQDELLW GAAWLHKASR
FcEG1       P--------Y SDHNCWERPE DMDTRRTVYK IDHNNPGSDV AGETAAALAA ASIVFRSRDP AYSRLLLNRA VKVFEFADTH RGAYSSS--L KNAVCPFYCD VNGFQDELLW GAAWLHKASR
AtGH9B13    A--------N KDHSCWERPE DMDTVRSVFK VDKNIPGSDV AAETAAALAA AAIVFRKSDP SYSKVLLKRA ISVFAFADKY RGTYSAG--L KPDVCPFYCS YSGYQDELLW GAAWLQKATK
ATGH9B4.1   P--------N GDHKCWERPE DMDTPRTVYS VSPSNPGSDV AAETAAALAA SSMVFRKVDP KYSRLLLATA KKVMQFAIQY RGAYSNS--L SSSVCPFYCS YSGYKDELLW GAAWLHRATN
ATGH9B4.2l  P--------N GDHKCWERPE DMDTPRTVYS VSPSNPGSDV AAETAAALAA SSMVFRKVDP KYSRLLLATA KKVMQFAIQY RGAYSNS--L SSSVCPFYCS YSGYKDELLW GAAWLHRATN
ATGH9B3     P--------N VDHKCWERPE DMDTPRTVYS VSASNPGSDV AAETAAALAA ASMVFRKVDS KYSRLLLATA KDVMQFAIQY QGAYSDS--L SSSVCPFYCS YSGYKDELMW GASWLLRATN
AtGH9B17    V--------Q PDHDCWERPE DMDTPRTAFA LDAPYPASDL AGEIAAALAA ASIAFKQANP KYSAILLNKA VQTFQYADSH RGSYTDNPGI KQAVCPFYCS VNGYKDELLW GAAWLRRATG
AtGH9B16    P--------T PDHQCWERPE DMDTPRTAYA LEAPKPASDL AGEIAAALAA ASIAFKRFDP RYSKLLLDNA LRTFD----- ---------- ---------- VNGYEDELLW GAAWLRRATG
AtGH9B18    P--------I ADHNCWERPE DMDTPRTAYA VNAPNPASEV AGETTAALSA ASIAFRSSDP GYSQTLLQNA VKTFQFADMY RGAYSSNDDI KNDVCPFYCD FNGFQDELLW GAAWLRKATG
AtGH9B15    G--------N SDHSCWERPE DMDTSRTLYS ISSSSPGSEA AGEAAAALAA ASLVFKSVDS TYSSTLLNHA KTLFEFADKY RGSYQAS--- ----CPFYCS YSGYQDELLW AAAWLYKATG
AtGH9B14    G--------N SDHSCWERPE DMDTPRTLYS ISSSSPGSEA AGEAAAALAA ASLVFKLVDS TYSSKLLNNA KSLFEFADKY RGSYQAS--- ----CPFYCS HSGYQDELLW AAAWLYKATG
AtGH9B7     P--------K VDHPCWERPE DMKEKRPLTK IDVDTPGTEV AAETAAAMAS ASLVFKDSDP TYSATLLKHA KQLFNFADTK RGSYSVN--- -IPEVQKFYN STGYGDELLW AASWLYHATE
AtGH9B5     P--------V TDHKCWDRPE TMTRKRTLTK IDTKTPGTEV AAETAAAMAA ASLVFKESDT KYSSTLLKHA KQLFDFADNN RGSYSVN--- -IPEVQSYYN STGYGDELLW AASWLYHATE
ATGH9A1     GNTDDGNTDP NDHYCWMRPE DMDYKRP--V TTCNGGCSDL AAEMAAALAS ASIVFKD-NK EYSKKLVHGA KVVYQFGRTR --RGRYS--A GTAESSKFYN SSMYWDEFIW GGAWMYYATG
ATGH9A3     GVSGRGSELH NDHYCWMRPE DIHYKRT--V SQCYSSCSDL AAEMAAALAS ASIVFKD-NR LYSKNLVHGA KTLYRFATTS --RNRYS--Q NGKESSKFYN SSMFEDELLW GGAWLYYATG
ATGH9A2     G--LRDSESP DDIYCWQKPE DMSYDRP--V LSSTS-AADL GAEVSAALAA ASIVFTD-KP DYAKKLKKGA ETLYPFFRSK SRRKRYS--D GQPTAQAFYN STSMFDEFMW AGAWLYYATG


                    370        380        390        400        410        420        430        440        450        460        470        480
            ....|....| ....|....| ....|....| ....|....| ....|....| ....|....| ....|....| ....|....| ....|....| ....|....| ....|....| ....|....|
AtGH9B9     DQIYLNYLTQ AS-------- GTGGPRSVFA WDDKFVGAQV LMAKLALER- KVG-SNGKIA EYKSMAEQFI CNCAQKGSN- -NVKKTPGGL LYFLPWNN-- LQYTTTASFV LSAYSKYLTK
AtGH9B10    DQIYLDYLTE AE-------- TGG-PRTVFA WDDKFVGAQV LVAKLALEG- KVE-SSEQIV EYKSMAEQFI CNCAQKGDN- -NVKKTPGGL LYFLPWNN-- LQYTTAATFV LSAYSKYLEA
AtGH9B11    DQIYLDYLTQ AS-------- GTGGPRTAFS WDDKFVGAQV LVAKLALEG- KVE-SNGKIA EYKSMAEQFI CNCAQKGSN- -NVKKTPGGL LYFLPWNN-- LQYTTAATFV LSAYSKYLTD
AtGH9B12    DQTYLDHLTQ AS-------- NSGGARSVFA WDDKFLGAQV LVAKLVFEG- KVK-NEGKMI EYKSMAEQFI CNCAQKGFN- -NVKKTPGGL LWFLPWDN-- LQYTATASFA LATYAKYLEA
AtGH9C3     DKYYLDYLGK NGDS----MG GTGWSMTEFG WDVKYAGVQT LVAKVLMQG- KGGEHTAVFE RYQQKAEQFM CSLLGKSTK- -NIKKTPGGL IFRQSWNN-- MQFVTSASFL ATVYSDYLSY
AtGH9C2     NQFYLDYLGR NGDA----MG GTGWSMTEFG WDVKYAGVQT LVAKFLMQG- KAGRHAPVFR KYQEKADSFM CSLLGKSSR- -NIQKTPGGL IFRQRWNN-- MQFVTSASFL TTVYSDYLTS
AtGH9B8     NEHYMSYVVD MAHQ----LG GLSWAMSEFS WDVKFAGVQL LASMLLKEE- KHKQHSKVLQ QYKSKADHYL CSILNKNING TNVQRTPAGL LYVRQWNN-- MQYVSTASFL LTVYSDHLRK
AtGH9C1     DQYYLKYAMD NSGY----MG GTGWGVKEFS WDNKYAGVQI LLSKILLEG- KGGIYTSTLK QYQTKADYFA CACLKKNGG- YNIQTTPGGL MYVREWNN-- LQYASAAAYL LAVYSDYLSA
AtGH9B6     KRVYREFIVK NQV-----IL RAGDTIHEFG WDNKHAGINV LVSKMV---- -LMGKAEYFQ SFKQNADEFI CSLLPGISH- PQVQYSQGGL LVKSGGSN-- MQHVTSLSFL LLTYSNYLSH
ATGH9B1     KRAYREFIVK NEV-----IL KAGDTINEFG WDNKHAGINV LISKEV---- -LMGKAEYFE SFKQNADGFI CSILPGISH- PQVQYSRGGL LVKTGGSN-- MQHVTSLSFL LLAYSNYLSH
FcEG1       RRQYREYIVR NEV-----IL RAGDTINEFG WDNKHAGINI LISKEV---- -LMGKADYFE SFKQNADGFI CSVLPGLAH- TQVQYSPGGL IFKPGGSN-- MQHVTSLSFL LLTYSNYLSH
AtGH9B13    NIKYLNYIKI NGQ-----IL GAAEYDNTFG WDNKHAGARI LLTKAF---- -LVQNVKTLH EYKGHADNFI CSVIPGAPF- SSTQYTPGGL LFKMADAN-- MQYVTSTSFL LLTYAKYLTS
ATGH9B4.1   DPYYTNFIKS ---------L GGGDQPDIFS WDNKYAGAYV LLSRRA---- -VLNKDNNFE LYKQAAENFM CKILPNSPS- SSTKYTKGGL MYKLPQSN-- LQYVTSITFL LTTYAKYMKS
ATGH9B4.2l  DPYYTNFIKS ---------L GGGDQPDIFS WDNKYAGAYV LLSRRA---- -VLNKDNNFE LYKQAAENFM CKILPNSPS- SSTKYTKGGL MYKLPQSN-- LQYVTSITFL LTTYAKYMKS
ATGH9B3     NPYYANFIKS ---------L GGGDQPDIFS WDNKYAGAYV LLSRRA---- -LLNKDSNFE QYKQAAENFI CKILPDSPS- SSTQYTQGGL MYKLPQSN-- LQYVTSITFL LTTYAKYMKA
AtGH9B17    EDSYLRYLVD NGQ-----AF GESSNYFEFG WDNKVGGVNV LVAKEV---- -LQNNVTAIA AYKDTAEKMM CSFLPETNG- PHMSYTPGGL IYKPGSTQ-- LQNTAALSFL LLTYADYLST
AtGH9B16    KDSYIKYLVE NRQ-----SF GSDSNYFEFG WDNKVGGVNV LVAKEV---- -FEKNVAAIA PYKDTAEKLM CSFFLETPG- AHMSYSPGGL LYKPGSSQ-- LQNTVALSFL LLTYANYLSK
AtGH9B18    DESYLNYIES NRE-----PF GANDNVDEFG WDNKVGGLNV LVSKEV---- -IEGNMYNLE AYKASAESFM CSLVPESSG- PHVEYTSAGL LYKPGGSQ-- LQHATTISFL LLVYAQYLSR
AtGH9B15    DKIYINYVIS NK-------- DWSQAVNEFS WDNKFVGAQA LLVSEF---- -YNG-ANDLA KFKSDVESFV CAMMPGSSS- QQIKPTPGGL LFIRDSSN-- LQYVTTATTV LFHYSKTLTK
AtGH9B14    EKSYLNYVIS NK-------- DWSKAINEFS WDNKFAGVQA LLASEF---- -YNG-ANDLE KFKTDVESFV CALMPGSSS- QQIKPTPGGI LFIRDSSN-- LQYVTTATTI LFYYSKTLTK
AtGH9B7     DKTYLDYVSN HGK-----EF ASFGNPTWFS WDNKLAGTQV LLSRLLFFK- KDLSGSKGLG NYRNTAKAVM CGLLPKSPT- STASRTNGGL IWVSEWNS-- MQQSVSSAFL ASLFSDYMLT
AtGH9B5     DQTYLDFVSE NGE-----EF GNFGSPSWFS WDNKLPGTHI LLSRLTFFK- KGLSGSKGLQ GFKETAEAVM CGLIPSSPT- ATSSRTDGGL IWVSEWNA-- LQHPVSSAFL ATLYSDYMLT
ATGH9A1     NVTYLNLITQ PTMAKHAGAF WGGPYYGVFS WDNKLAGAQL LLSRLRLFLS PGYPYEEILR TFHNQTSIVM CSYLPIFNK- --FNRTNGGL IELNHGAPQP LQYSVNAAFL ATLYSDYLDA
ATGH9A3     NVTYLERVTS HHMAEKAGAF GNSPYYGVFS WDNKLPGAQL LLTRMRLFLS PGYPYEDMLS EFHNQTGRVM CSYLPYYKK- --FNRTNGGL IQLNHGAPQP LQYVANAAFL AALFSDYLEA
ATGH9A2     NKTYIQFATT PSVPQTAKAF ANRPELMVPS WNNKLPGAML LMTRYRLFLN PGFPYENMLN RYHNATGITM CAYLKQYNV- --FNRTSGGL MQLNLGKPRP LEYVAHASFL ASLFADYLNS

                    490        500        510        520        530        540        550        560        570        580        590        600
            ....|....| ....|....| ....|....| ....|....| ....|....| ....|....| ....|....| ....|....| ....|....| ....|....| ....|....| ....|....|
AtGH9B9     AKASIQCPKG -ALQASDLLQ IARSQVDYIL GSNPQKMSYM VGVGTNYPKK PHHRAASIVS IRKDKTPVTC SGGYDKWYNN PAPNPNVLMG ALVGGPNEND VYGDERSNFQ QAEPATVTVA
AtGH9B10    AKASIDCPDG -ALQASDLLQ VARSQVDYIL GSNPQKMSYM VGVGTNYPKK PHHRAASIVS IRQDKTPVTC SGGYDKWYNN PAPNPNVLAG AVVGGPDDND VYGDERSNFQ QAEPATVTTA
AtGH9B11    AKASIQCPNG -ALQASDLLD LARSQVDYIL GSNPQNMSYM VGVGTNYPKK PHHRAASIVS ITKDKTPVTC SEGFDAWFNN PAPNPNVLMG AVVGGPNDND VYGDERTDYQ HAEPAPATAA
AtGH9B12    AQTSIQCPNG DVLQASDLLN LARAQVDYIL GSNPKKMSYM VGYGTNYPKR PHHRGASIVS IKNDPKPVTC NGGFEAWYNN PKPNPNLLVG AIVGGPDEYD AYGDERSDFQ HGEPDTVTVA
AtGH9C3     SKRDLLCSQG -NISPSQLLE FSKSQVDYIL GDNPRATSYM VGYGENYPRQ VHHRGSSIVS FNVDQKFVTC RGGYATWFSR KGSDPNVLTG ALVGGPDAYD NFADQRDNYE QTEPATYNNA
AtGH9C2     SRSNLRCAAG -NVAPSQLLS FAKSQVDYIL GDNPRATSYM VGYGNNFPQR VHHRGSSIVS VKVDRTFVTC RGGYATWFSR KGSDPNLLTG AIVGGPDAYD NFADRRDNYE QTEPATYNNA
AtGH9B8     SNTDLECHEG -TVTPDEMLG FAKSQIDYIL GSNPMETSYL VGYGPKYPIR VHHRGASIAS FKEHKGFIGC TQGYDNWYGR SEPNPSVLVG ALVGGPDHQD DFDDRRGNYV QTEACTYNTA
AtGH9C1     ANAKLNCPDG -LVQPQGLLD FARSQADYIL GKNRQGMSYV VGYGPKYPIR VHHRGSSIPS IFAQRSSVSC VQGFDSWYRR SQGDPNVIYG ALVGGPDEND NYSDDRSNYE QSEPTLSGTA
AtGH9B6     A-NKVVPCGE FTASPALLRQ VAKRQVDYIL GDNPMKMSYM VGYGSRFPQK IHHRGSSVPS VVDHPDRIGC KDGSRYFFSN N-PNPNLLIG AVVGGPNITD DFPDSRPYFQ LTEPTTYINA
ATGH9B1     A-KKVVPCGE LTASPSLLRQ IAKRQVDYIL GDNPMGLSYM VGYGQKFPRR IHHRGSSVPS VSAHPSHIGC KEGSRYFLSP N-PNPNLLVG AVVGGPNVTD AFPDSRPYFQ QSEPTTYINA
FcEG1       A-NKNVPCGM TSASPAFLKQ LAKRQVDYIL GDNPLRMSYM VGYGPRYPQR IHHRGSSLPS VQAHPARIGC KAGSRYFLSP N-PNPNKLVG AVVGGPNSSD AFPDSRPYFQ ESEPTTYINA
AtGH9B13    A-KTVVHCGG SVYTPGRLRS IAKRQVDYLL GDNPLRMSYM VGYGPKFPRR IHHRGSSLPC VASHPAKIQC HQGFAIMNSQ S-PNPNFLVG AVVGGPDQHD RFPDERSDYE QSEPATYINS
ATGH9B4.1   T-KQTFNCGN SLIVPNALIN LSKRQVDYVL GVNPMKMSYM VGFSSNFPKR IHHRGSSLPS RAVRSNSLGC NGGFQSFRTQ N-PNPNILTG AIVGGPNQND EYPDQRDDYT RSEPATYINA
ATGH9B4.2l  T-KQTFNCGN SLIVPNALIN LSKRQVDYVL GVNPMKMSYM VGFSSNFPKR IHHRGSSLPS RAVRSNSLGC NGGFQSFRTQ N-PNPNILTG AIVGGPNQND EYPDQRDDYT RSEPATYINA
ATGH9B3     T-KHTFNCGS SVIVPNALIS LSKRQVDYIL GDNPIKMSYM VGFSSNFPKR IHHRASSLPS HALRSQSLGC NGGFQSFYTQ N-PNPNILTG AIVGGPNQND GYPDQRDDYS HAEPATYINA
AtGH9B17    S-SQQLNCGN LKFQPDSLRR IVKRQVDYVL GDNPMKLSYM IGYGERYPGL IHHRGSSIPS VTVHPAAFGC IAGWNIFSSP N-PNPNILIG AVIGGPDVDD RFIGGRTNAS ETEPTTYINA
AtGH9B16    S-SQQP---- ---------- ------LQIL STTPLW---- ---------- ---------- ---------- ---------- ---------- ---------- ---------- ----------
AtGH9B18    S-SLSLNCGT LTVPPDYLRR LAKKQVDYIL GNNPMGLSYM VGYGERYPKR IHHRGSSLPS IVDHPEAIRC KDGSVYFNST E-PNPNVLIG AVVGGPGEDD MYDDDRSDFR KSEPTTYINA
AtGH9B15    AGVGSIQCGS TKFTVSQIRN FAKSQVDYIL GNNPMKMSYM VGFGTKYPTQ PHHRGSSLPS IQSKPEKIDC NGGYSYYNSD T-PNPNVHIG AIVGGPNSSD QYSDKKSDYS HAEPTTYINA
AtGH9B14    AGVGSIQCGS TQFTVSQIRN FAKSQVDYIL GNNPLKMSYM VGFGTKYPTQ PHHRGSSLPS IQSKPEKIDC NGGFSYYNFD T-PNPNVHTG AIVGGPNSSD QYSDKRTDYS HAEPTTYINA
AtGH9B7     SRIHKISCDG KIFKATELRD FAKSQADYML GKNPLGTSFV VGYGDKYPQF VHHRGASIP- ---ADATTGC LDGFKWFNST K-PNPNIAYG ALVGGPFFNE TFTDSRENPM QNEPTTYNNA
AtGH9B5     SGVKELSCSD QSFKPSDLRK FARSQADYML GKNPEKMSYL VGYGEKYPEF VHHRGASIP- ---ADATTGC KDGFKWLNSD E-PNPNVAYG ALVGGPFLND TFIDARNNSM QNEPSTYNSA
ATGH9A1     ADTPGWYCGP NFYSTSVLRD FARSQIDYIL GKNPRKMSYV VGFGTKYPRH VHHRG---AS IPKNKVKYNC KGGWKWRDSK K-PNPNTIEG AMVAGPDKRD GYRDVRMNYN YTEPTLAGNA
ATGH9A3     ADTPGWYCGP NFYTTEFLRN FSRSQIDYIL GKNPRKMSYV VGYGQRYPKQ VHHRG---AS IPKN-MKETC TGGFKWKKSK K-NNPNAING AMVAGPDKHD GFHDIRTNYN YTEPTLAGNA
ATGH9A2     TGVPGWYCGP TFVENHVLKD FAQSQIDYIL GDNPLKMSYV VGFGKKFPRR VHHRG---AT IPNDKKRRSC REGLKYRDTK N-PNPNNITG AMVGGPNKFD EFHDLRNNYN ASEPTLSGNA


                    610        620        630        640        650        660        670        680        690        700        710        720
            ....|....| ....|....| ....|....| ....|....| ....|....| ....|....| ....|....| ....|....| ....|....| ....|....| ....|....| ....|....|
AtGH9B9     PFVGVLAAVF ---------- ---------- ---------- ---------- ---------- ---------- ---------- ---------- ---------- ---------- ----------
AtGH9B10    PLVGVLALVF ---------- ---------- ---------- ---------- ---------- ---------- ---------- ---------- ---------- ---------- ----------
AtGH9B11    PFVGVLAAVA ---------- ---------- ---------- ---------- ---------- ---------- ---------- ---------- ---------- ---------- ----------
AtGH9B12    PLLGVLAAIA ---------- ---------- ---------- ---------- ---------- ---------- ---------- ---------- ---------- ---------- ----------
AtGH9C3     PLLGVLARLI SGSTGFDQLL PGVSPTPSPV IIKPAPVPQR KPTKPPAASS PSPITISQKM TNSWKNEGKV YYRYSTILTN RSTKTLKILK ISITKLYGPI WGVTKT--GN SFSFPSWMQS
AtGH9C2     PLLGVLARLS SGHSGYSQFL P---VVPAPV VRRPMPIRRP KVTTPVRASG --PVAIVQKI TSSWVSKGRT YYRYSTTVIN KSSRPLKSLN LSIKNLYGPI WGLSRS--GN SFGLPSWMHS
AtGH9B8     PLVGVFARLI ELEE------ ---------- ---------- ---------- -------QKL EEEDVSLVAT YKR------- ---------- ---------- ---------- ----------
AtGH9C1     PLVGLFAKLY GGSLGSYGGG S---YKPYET TKPAASSYKA TPTTYSPKQS GAQIEFLHSI TSNWIAGNTR YYRHKVIIKN NSQKPISDLK LKIEDLSGPI WGLNPTGQKY TYQLPQWQKT
AtGH9B6     PLLGLLGYFS AHY------- ---------- ---------- ---------- ---------- ---------- ---------- ---------- ---------- ---------- ----------
ATGH9B1     PLVGLLGYFS AHSTWR---- ---------- ---------- ---------- ---------- ---------- ---------- ---------- ---------- ---------- ----------
FcEG1       PLVGLLSYFA AHY------- ---------- ---------- ---------- ---------- ---------- ---------- ---------- ---------- ---------- ----------
AtGH9B13    PLVGALAYFA HAYGQL---- ---------- ---------- ---------- ---------- ---------- ---------- ---------- ---------- ---------- ----------
ATGH9B4.1   AFVGPLAYFA ASRSP----- ---------- ---------- ---------- ---------- ---------- ---------- ---------- ---------- ---------- ----------
ATGH9B4.2l  AFVGPLAYFA ASRSP----- ---------- ---------- ---------- ---------- ---------- ---------- ---------- ---------- ---------- ----------
ATGH9B3     AFVGPLAYFA AGRST----- ---------- ---------- ---------- ---------- ---------- ---------- ---------- ---------- ---------- ----------
AtGH9B17    PFVGVFAYFK SNPNFS---- ---------- ---------- ---------- ---------- ---------- ---------- ---------- ---------- ---------- ----------
AtGH9B16    ---------- ---------- ---------- ---------- ---------- ---------- ---------- ---------- ---------- ---------- ---------- ----------
AtGH9B18    PFVGVLAYFA ANPGSS---- ---------- ---------- ---------- ---------- ---------- ---------- ---------- ---------- ---------- ----------
AtGH9B15    AFIGPVAALI SSSG------ ---------- ---------- ---------- ---------- ---------- ---------- ---------- ---------- ---------- ----------
AtGH9B14    AFIGSVAALI SSS------- ---------- ---------- ---------- ---------- ---------- ---------- ---------- ---------- ---------- ----------
AtGH9B7     LLVGLLSSLV TTSSTLQSLK ---------- ---------- ---------- ---------- ---------- ---------- ---------- ---------- ---------- ----------
AtGH9B5     LVVGLLSSLV TTSSSVESF- ---------- ---------- ---------- ---------- ---------- ---------- ---------- ---------- ---------- ----------
ATGH9A1     GLVAALVALS GEEEATGKID KNTIFSAVPP LFPTPPPPPA PWKP------ ---------- ---------- ---------- ---------- ---------- ---------- ----------
ATGH9A3     GLVAALVALS GEK-AVGGID KNTMFSAVPP LVMATPPPPA PWTP------ ---------- ---------- ---------- ---------- ---------- ---------- ----------
ATGH9A2     GLVAALVSLT SSG--GQQID KNTMFNSVPP LYSPTPPPPK AWKP------ ---------- ---------- ---------- ---------- ---------- ---------- ----------


                    730        740
            ....|....| ....|....| ....|...
AtGH9B9     ---------- ---------- --------
AtGH9B10    ---------- ---------- --------
AtGH9B11    ---------- ---------- --------
AtGH9B12    ---------- ---------- --------
AtGH9C3     LPSGKSMEFV YIHSASPADV LVSNYSLE
AtGH9C2     LPSGKSLEFV YIHSTTPANV AVSSYTLA
AtGH9B8     ---------- ---------- --------
AtGH9C1     LRAGQAYDFV YVQGGPQAKV SVLSYN--
AtGH9B6     ---------- ---------- --------
ATGH9B1     ---------- ---------- --------
FcEG1       ---------- ---------- --------
AtGH9B13    ---------- ---------- --------
ATGH9B4.1   ---------- ---------- --------
ATGH9B4.2l  ---------- ---------- --------
ATGH9B3     ---------- ---------- --------
AtGH9B17    ---------- ---------- --------
AtGH9B16    ---------- ---------- --------
AtGH9B18    ---------- ---------- --------
AtGH9B15    ---------- ---------- --------
AtGH9B14    ---------- ---------- --------
AtGH9B7     ---------- ---------- --------
AtGH9B5     ---------- ---------- --------
ATGH9A1     ---------- ---------- --------
ATGH9A3     ---------- ---------- --------
ATGH9A2     ---------- ---------- --------
```

**Figure S1. Comparison of FaEG1 sequence from *Fragaria* × *ananassa* with their orthologs.** Multiple alignment of FaEG1 sequence and othologs sequences from Arabidopsis thaliana. Gaps are indicated by dashes. Black is 100% identity and then becomes more white with increasing amino acid residue diversity.

**Figure S2. Comparison of FaEG1 sequence from *Fragaria* × *ananassa* with the 1KS8 sequence used as template.** Sequence alignment of FaEG1 sequence and homolog sequence with PDB code 1KS8. Gaps are indicated by dashes. Asterisk is 100% identity and residue similarity are indicated with “:” or “.”.

**Figure S3. RMSD of the protein ligand complex.** In blue line it shows the RMSD of the FaEG1-XXFGXXFG complex, in red line it shows the RMSD of the FaEG1-XXXGXXXG complex, and in Green line it shows the FaEG1-cellulose complex.
