# Supplementary material for: Molecular Insights into FaEG1, a Strawberry Endoglucanase Enzyme Expressed during Strawberry Fruit Ripening
Source: Plants (Basel). 2019 May 28;8(6):140. doi: 10.3390/plants8060140 (PMC6631567; doi:10.3390/plants8060140)
Supplement: Supplementary file 1 [file plants-08-00140-s001.zip › plants-501458-suppl-final/Table S1.doc]

**Table S1. Sequence identity matrix.**

|  | **AtGH9B9** | **AtGH9B10** | **AtGH9B11** | **AtGH9B12** | **AtGH9C3** | **AtGH9C2** | **AtGH9B8** | **AtGH9C1** | **AtGH9B6** | **ATGH9B1** | **FaEG1** | **AtGH9B13** | **ATGH9B4.1** | **ATGH9B4.2l** | **ATGH9B3** | **AtGH9B17** | **AtGH9B16** | **AtGH9B18** | **AtGH9B15** | **AtGH9B14** | **AtGH9B7** | **AtGH9B5** | **ATGH9A1** | **ATGH9A3** | **ATGH9A2** |
| --- | --- | --- | --- | --- | --- | --- | --- | --- | --- | --- | --- | --- | --- | --- | --- | --- | --- | --- | --- | --- | --- | --- | --- | --- | --- |
| AtGH9B9 | ID | 0,908 | 0,869 | 0,733 | 0,397 | 0,4 | 0,454 | 0,406 | 0,405 | 0,413 | 0,404 | 0,396 | 0,425 | 0,34 | 0,421 | 0,391 | 0,303 | 0,401 | 0,462 | 0,438 | 0,368 | 0,4 | 0,294 | 0,283 | 0,272 |
| AtGH9B10 | 0,908 | ID | 0,859 | 0,729 | 0,403 | 0,4 | 0,452 | 0,409 | 0,413 | 0,415 | 0,408 | 0,396 | 0,417 | 0,334 | 0,417 | 0,391 | 0,299 | 0,395 | 0,45 | 0,43 | 0,379 | 0,404 | 0,291 | 0,286 | 0,261 |
| AtGH9B11 | 0,869 | 0,859 | ID | 0,745 | 0,4 | 0,402 | 0,444 | 0,403 | 0,405 | 0,409 | 0,406 | 0,412 | 0,429 | 0,342 | 0,423 | 0,391 | 0,303 | 0,405 | 0,462 | 0,446 | 0,372 | 0,39 | 0,291 | 0,285 | 0,267 |
| AtGH9B12 | 0,733 | 0,729 | 0,745 | ID | 0,385 | 0,395 | 0,428 | 0,404 | 0,415 | 0,415 | 0,41 | 0,412 | 0,435 | 0,34 | 0,423 | 0,387 | 0,296 | 0,411 | 0,446 | 0,436 | 0,375 | 0,39 | 0,29 | 0,291 | 0,27 |
| AtGH9C3 | 0,397 | 0,403 | 0,4 | 0,385 | ID | 0,752 | 0,466 | 0,487 | 0,351 | 0,36 | 0,358 | 0,346 | 0,341 | 0,273 | 0,337 | 0,345 | 0,236 | 0,34 | 0,364 | 0,366 | 0,342 | 0,343 | 0,268 | 0,264 | 0,241 |
| AtGH9C2 | 0,4 | 0,4 | 0,402 | 0,395 | 0,752 | ID | 0,482 | 0,483 | 0,361 | 0,357 | 0,358 | 0,35 | 0,343 | 0,276 | 0,34 | 0,337 | 0,237 | 0,337 | 0,363 | 0,366 | 0,327 | 0,324 | 0,262 | 0,263 | 0,242 |
| AtGH9B8 | 0,454 | 0,452 | 0,444 | 0,428 | 0,466 | 0,482 | ID | 0,431 | 0,388 | 0,4 | 0,402 | 0,394 | 0,365 | 0,291 | 0,371 | 0,38 | 0,266 | 0,393 | 0,422 | 0,408 | 0,387 | 0,4 | 0,305 | 0,293 | 0,256 |
| AtGH9C1 | 0,406 | 0,409 | 0,403 | 0,404 | 0,487 | 0,483 | 0,431 | ID | 0,314 | 0,331 | 0,324 | 0,328 | 0,32 | 0,258 | 0,325 | 0,316 | 0,225 | 0,319 | 0,367 | 0,363 | 0,335 | 0,335 | 0,273 | 0,27 | 0,24 |
| AtGH9B6 | 0,405 | 0,413 | 0,405 | 0,415 | 0,351 | 0,361 | 0,388 | 0,314 | ID | 0,847 | 0,76 | 0,558 | 0,528 | 0,426 | 0,52 | 0,514 | 0,364 | 0,557 | 0,45 | 0,447 | 0,349 | 0,363 | 0,274 | 0,267 | 0,243 |
| ATGH9B1 | 0,413 | 0,415 | 0,409 | 0,415 | 0,36 | 0,357 | 0,4 | 0,331 | 0,847 | ID | 0,787 | 0,569 | 0,541 | 0,448 | 0,525 | 0,516 | 0,366 | 0,569 | 0,456 | 0,45 | 0,359 | 0,375 | 0,275 | 0,262 | 0,246 |
| **FaEG1** | **0,404** | **0,408** | **0,406** | **0,41** | **0,358** | **0,358** | **0,402** | **0,324** | **0,76** | **0,787** | **ID** | **0,573** | **0,527** | **0,43** | **0,517** | **0,532** | **0,367** | **0,574** | **0,466** | **0,462** | **0,368** | **0,371** | **0,267** | **0,267** | **0,245** |
| AtGH9B13 | 0,396 | 0,396 | 0,412 | 0,412 | 0,346 | 0,35 | 0,394 | 0,328 | 0,558 | 0,569 | 0,573 | ID | 0,535 | 0,442 | 0,537 | 0,475 | 0,328 | 0,511 | 0,448 | 0,445 | 0,363 | 0,376 | 0,283 | 0,281 | 0,266 |
| ATGH9B4.1 | 0,425 | 0,417 | 0,429 | 0,435 | 0,341 | 0,343 | 0,365 | 0,32 | 0,528 | 0,541 | 0,527 | 0,535 | ID | 0,826 | 0,871 | 0,474 | 0,323 | 0,521 | 0,471 | 0,475 | 0,357 | 0,379 | 0,277 | 0,264 | 0,268 |
| ATGH9B4.2l | 0,34 | 0,334 | 0,342 | 0,34 | 0,273 | 0,276 | 0,291 | 0,258 | 0,426 | 0,448 | 0,43 | 0,442 | 0,826 | ID | 0,737 | 0,385 | 0,233 | 0,428 | 0,388 | 0,388 | 0,297 | 0,318 | 0,217 | 0,207 | 0,209 |
| ATGH9B3 | 0,421 | 0,417 | 0,423 | 0,423 | 0,337 | 0,34 | 0,371 | 0,325 | 0,52 | 0,525 | 0,517 | 0,537 | 0,871 | 0,737 | ID | 0,452 | 0,308 | 0,498 | 0,471 | 0,477 | 0,355 | 0,375 | 0,275 | 0,261 | 0,257 |
| AtGH9B17 | 0,391 | 0,391 | 0,391 | 0,387 | 0,345 | 0,337 | 0,38 | 0,316 | 0,514 | 0,516 | 0,532 | 0,475 | 0,474 | 0,385 | 0,452 | ID | 0,525 | 0,603 | 0,417 | 0,419 | 0,338 | 0,354 | 0,263 | 0,26 | 0,243 |
| AtGH9B16 | 0,303 | 0,299 | 0,303 | 0,296 | 0,236 | 0,237 | 0,266 | 0,225 | 0,364 | 0,366 | 0,367 | 0,328 | 0,323 | 0,233 | 0,308 | 0,525 | ID | 0,442 | 0,3 | 0,298 | 0,233 | 0,24 | 0,185 | 0,182 | 0,171 |
| AtGH9B18 | 0,401 | 0,395 | 0,405 | 0,411 | 0,34 | 0,337 | 0,393 | 0,319 | 0,557 | 0,569 | 0,574 | 0,511 | 0,521 | 0,428 | 0,498 | 0,603 | 0,442 | ID | 0,473 | 0,465 | 0,348 | 0,377 | 0,257 | 0,253 | 0,234 |
| AtGH9B15 | 0,462 | 0,45 | 0,462 | 0,446 | 0,364 | 0,363 | 0,422 | 0,367 | 0,45 | 0,456 | 0,466 | 0,448 | 0,471 | 0,388 | 0,471 | 0,417 | 0,3 | 0,473 | ID | 0,916 | 0,373 | 0,4 | 0,293 | 0,295 | 0,273 |
| AtGH9B14 | 0,438 | 0,43 | 0,446 | 0,436 | 0,366 | 0,366 | 0,408 | 0,363 | 0,447 | 0,45 | 0,462 | 0,445 | 0,475 | 0,388 | 0,477 | 0,419 | 0,298 | 0,465 | 0,916 | ID | 0,377 | 0,394 | 0,294 | 0,293 | 0,272 |
| AtGH9B7 | 0,368 | 0,379 | 0,372 | 0,375 | 0,342 | 0,327 | 0,387 | 0,335 | 0,349 | 0,359 | 0,368 | 0,363 | 0,357 | 0,297 | 0,355 | 0,338 | 0,233 | 0,348 | 0,373 | 0,377 | ID | 0,709 | 0,333 | 0,323 | 0,304 |
| AtGH9B5 | 0,4 | 0,404 | 0,39 | 0,39 | 0,343 | 0,324 | 0,4 | 0,335 | 0,363 | 0,375 | 0,371 | 0,376 | 0,379 | 0,318 | 0,375 | 0,354 | 0,24 | 0,377 | 0,4 | 0,394 | 0,709 | ID | 0,31 | 0,308 | 0,288 |
| ATGH9A1 | 0,294 | 0,291 | 0,291 | 0,29 | 0,268 | 0,262 | 0,305 | 0,273 | 0,274 | 0,275 | 0,267 | 0,283 | 0,277 | 0,217 | 0,275 | 0,263 | 0,185 | 0,257 | 0,293 | 0,294 | 0,333 | 0,31 | ID | 0,77 | 0,545 |
| ATGH9A3 | 0,283 | 0,286 | 0,285 | 0,291 | 0,264 | 0,263 | 0,293 | 0,27 | 0,267 | 0,262 | 0,267 | 0,281 | 0,264 | 0,207 | 0,261 | 0,26 | 0,182 | 0,253 | 0,295 | 0,293 | 0,323 | 0,308 | 0,77 | ID | 0,537 |
| ATGH9A2 | 0,272 | 0,261 | 0,267 | 0,27 | 0,241 | 0,242 | 0,256 | 0,24 | 0,243 | 0,246 | 0,245 | 0,266 | 0,268 | 0,209 | 0,257 | 0,243 | 0,171 | 0,234 | 0,273 | 0,272 | 0,304 | 0,288 | 0,545 | 0,537 | ID |
